# ^18^F-FDG PET/CT Maximum Tumor Dissemination (Dmax) in Lymphoma: A New Prognostic Factor?

**DOI:** 10.3390/cancers15092494

**Published:** 2023-04-26

**Authors:** Domenico Albano, Giorgio Treglia, Francesco Dondi, Anna Calabrò, Alessio Rizzo, Salvatore Annunziata, Luca Guerra, Silvia Morbelli, Alessandra Tucci, Francesco Bertagna

**Affiliations:** 1 Division of Nuclear Medicine, Università degli Studi di Brescia, ASST Spedali Civili di Brescia, 25123 Brescia, Italy; f.dondi@outlook.com (F.D.); anna_calabro@hotmail.it (A.C.); francesco.bertagna@unibs.it (F.B.); 2 Clinic of Nuclear Medicine, Imaging Institute of Southern Switzerland, Ente Ospedaliero Cantonale, 6501 Bellinzona, Switzerland; giorgio.treglia@eoc.ch; 3 Faculty of Biology and Medicine, University of Lausanne, 1011 Lausanne, Switzerland; 4 Faculty of Biomedical Sciences, Università della Svizzera Italiana, 6900 Lugano, Switzerland; 5 Department of Nuclear Medicine, Candiolo Cancer Institute, FPO-IRCCS, 10060 Turin, Italy; alessio.rizzo@ircc.it; 6 Unità di Medicina Nucleare, TracerGLab, Dipartimento di Diagnostica per Immagini, Radioterapia Oncologica ed Ematologia, Fondazione Policlinico Universitario A. Gemelli, IRCCS, 00168 Rome, Italy; salvatore.annunziata@policlinicogemelli.it; 7 Nuclear Medicine Division, Ospedale San Gerardo, 20900 Monza, Italy; luca.guerra@unimib.it; 8 Nuclear Medicine Unit, IRCCS Ospedale Policlinico San Martino, 16132 Genoa, Italy; silviadaniela.morbelli@hsanmartino.it; 9 Hematology, ASST Spedali Civili, 25123 Brescia, Italy; alessandra.tucci@asst-spedalicivili.it

**Keywords:** PET/CT, lymphoma, ^18^F-FDG, Dmax, nuclear medicine

## Abstract

**Simple Summary:**

The prognostic stratification of patients affected by lymphomas is critical, and many positron-emission tomography (PET) metabolic parameters such as SUV, MTV, and TLG have been studied for this purpose. However, the lack of technical standardization in their measurements strongly affects their clinical power and potential integration into clinical practice. Dmax is a new biomarker that measures the distance between the two farthest hypermetabolic PET lesions and seems to show high accuracy as a prognostic factor in patients with lymphomas. The aim of this systematic review was to provide an evidence-based overview of the role of Dmax in lymphomas.

**Abstract:**

Recently, several studies introduced the potential prognostic usefulness of maximum tumor dissemination (Dmax) measured by 2-deoxy-2-fluorine-18-fluoro-D-glucose positron-emission tomography/computed tomography (^18^F-FDG PET/CT). Dmax is a simple three-dimensional feature that represents the maximal distance between the two farthest hypermetabolic PET lesions. A comprehensive computer literature search of PubMed/MEDLINE, Embase, and Cochrane libraries was conducted, including articles indexed up to 28 February 2023. Ultimately, 19 studies analyzing the value of ^18^F-FDG PET/CT Dmax in patients with lymphomas were included. Despite their heterogeneity, most studies showed a significant prognostic role of Dmax in predicting progression-free survival (PFS) and overall survival (OS). Some articles showed that the combination of Dmax with other metabolic features, such as MTV and interim PET response, proved to better stratify the risk of relapse or death. However, some methodological open questions need to be clarified before introducing Dmax into clinical practice.

## 1. Introduction

The prognostic stratification of patients affected by lymphomas is crucial for treatment selection and subsequent follow-up. An early and accurate identification of the patients at a high risk of treatment failure and/or relapse may guide a more appropriate treatment or follow-up choice; this system is called a “risk-adapted strategy”, and needs the presence of robust and reproducible biomarkers.

Nowadays, 2-deoxy-2-fluorine-18-fluoro-D-glucose positron-emission tomography/computed tomography (^18^F-FDG PET/CT) is considered to be the best imaging tool for the staging and treatment response evaluation of ^18^F-FDG avid lymphomas, which include Hodgkin lymphoma (HL), diffuse large B-cell lymphoma (DLBCL), and follicular lymphoma (FL) [1,2]. Its usefulness in other lymphoma variants remains unclear [3,4,5].

Moreover, several PET baseline semi-quantitative parameters and metabolic response statuses after therapy have been demonstrated to be optimal prognostic variables in ^18^F-FDG avid lymphomas [6,7].

Increasing evidence has suggested that baseline metabolic tumor volume (MTV) and total lesion glycolysis (TLG) may be valid predictors of the patient outcome, both in HL and NHL, but the lack of technical standardization in their measurements strongly affects their clinical power and potential integration into clinical practice [6,7].

For this reason, other metabolic imaging biomarkers have been investigated such as texture features [8] and sarcopenic parameters [9], with promising results. Another recently analyzed feature was the maximum tumor dissemination (Dmax), which is defined as the maximal distance between the two farthest hypermetabolic lesions using ^18^F-FDG PET/CT [10]. In other words, this is a parameter that may express the dissemination/spread of disease in the whole body [11]. However, despite promising results, a shared consensus about the best way to measure Dmax is unclear.

The aim of this systematic review was to investigate the published data on the role of ^18^F-FDG Dmax in patients affected by lymphomas to clarify its potential clinical and prognostic role in these diseases.

## 2. Materials and Methods

### 2.1. Protocol

This systematic review was conducted according to the PRISMA statement [12], and the review question was to investigate the potential role of Dmax in patients affected by lymphomas. The PRISMA checklist is available in Supplementary Table S1. In agreement with the population, intervention, comparator, and outcomes (PICO) framework, two reviewers (D.A and F.D.) accomplished a literature search, establishing the criteria for the eligibility of the studies found in the literature search. The criteria were patients affected by lymphomas (population) undergoing a PET with ^18^F-FDG, including an analysis of Dmax (intervention) compared or not with other PET/CT features (comparator); the predetermined outcomes were the evaluation of the potential clinical and prognostic role of Dmax in patients with lymphomas (outcome).

### 2.2. Literature Search Strategy

Taking into account the review query, a comprehensive literature search of Scopus, PubMed/MEDLINE, Embase, and Cochrane library databases was conducted to find relevant published articles on the role of Dmax in patients affected by lymphomas. The ClinicalTrials.gov database was also used to look for ongoing studies (access date: 28 February 2023). A search algorithm based on a combination of the following terms was used: (a) “Dmax” OR “dissemination” OR “distance” AND (b) “lymphoma” OR “lymphoproliferative” AND (c) “PET” OR “positron”. No limitation regarding the study period date was applied, and the search was updated until 28 February 2023. Only articles in the English language were selected. To enlarge our research, references of the retrieved articles were also screened to search for additional records.

### 2.3. Study Selection Process

Original papers reporting data about the role of ^18^F-FDG Dmax in lymphomas were eligible for inclusion in this systematic review. Meta-analyses, reviews, editorials, comments, and letters concerning the selected topic as well as original papers not in the field of interest (including preclinical studies) and small case series (less than 10 patients included) or case reports concerning the analyzed topic were excluded from the systematic review. Two researchers (D.A. and F.D.) independently reviewed the titles and abstracts of the records, and independently reviewed the full-text version of the articles to evaluate their suitability. In the case of a disagreement, a third opinion (F.B.) was involved in the selection process to settle any disagreement.

### 2.4. Data Collection Process and Extraction

For every included study, data were collected concerning the basic study features (first author name, year of publication, country, and study design), the main clinical patient features (number of patients, age, gender, and type of lymphomas), technical variables (PET device used, metabolic features analyzed, and software used), and the main findings. The main findings of the papers analyzed in this review are described in the Results section. Two authors (D.A. and F.D.) independently performed the data collection and extraction.

### 2.5. Quality Assessment

The quality assessment included a valuation of both the risk of bias and applicability concerns using a QUADAS-2 evaluation [13]. Two reviewers (D.A. and F.D.) independently assessed the quality of the studies included in the systematic review. Four fields (patient selection, index test, reference standard, and flow and timing) were assessed regarding the risk of bias, and three domains were evaluated regarding applicability (patient selection, index test, and reference standard). Any disagreement between the authors during the quality assessment was submitted to and solved by a third researcher (F.B.).

### 2.6. Statistical Analysis

Due to the heterogeneity of the available studies (different types of lymphomas), we planned a systematic review (qualitative synthesis) without a meta-analysis (quantitative synthesis). Therefore, a statistical analysis (pooled analysis) was not performed. Unfortunately, we could not combine all research data through a pooled analysis to calculate the best cut-off value of Dmax. There were two reasons: the different cut-off values of Dmax used by the different authors, and the inability to recalculate each Dmax value for each patient included.

Progression-free survival (PFS) and overall survival (OS) were defined according to data provided by the authors of the original articles as the time interval from the initial diagnosis until disease relapse, progression, death, or the last follow-up for PFS, and as a time interval from the initial diagnosis until death or the last follow-up for OS.

## 3. Results

### 3.1. Literature Search and Study Selection

**Figure 1:**
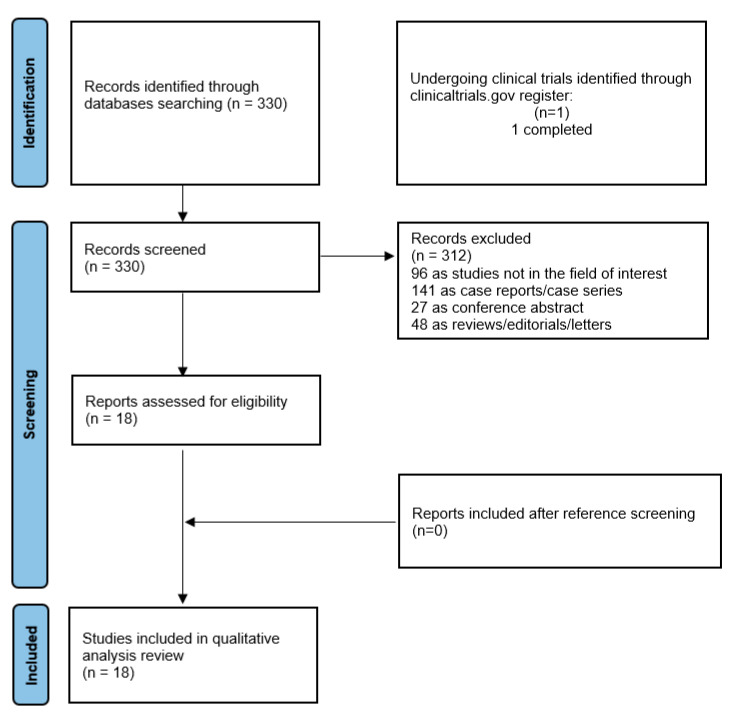
Comprehensive overview of the study selection process for the systematic review.

The literature search was last updated on 28 February 2023 and revealed a total of 330 records. Based on the inclusion and exclusion criteria mentioned above, 312 records were excluded (96 as not in the field of interest; 48 as reviews, editorials, or letters; 141 as case reports or case series; and 27 as conference abstracts). Eighteen remaining records were eligible for inclusion in the systematic review (qualitative synthesis) after a full-text assessment [10,14,15,16,17,18,19,20,21,22,23,24,25,26,27,28,29,30]. No additional records were assessed as suitable for inclusion after screening the references of these articles. Figure 1 summarizes the study selection process.

### 3.2. Study Characteristics

The main features of the 18 included studies in the systematic review are described in Table 1, Table 2 and Table 3 [10,14,15,16,17,18,19,20,21,22,23,24,25,26,27,28,29,30]. Regarding general study information (Table 1), all articles were published in the past four years (2020–2023) in Europe, Asia, and the USA. All studies but three had a retrospective design, and seven [14,19,20,21,22,24,25] of these articles declared funding in their text.

Regarding the patients’ key characteristics (Table 2), the total number of recruited patients was 3136, ranging from 30 to 382 in the different studies (median age from 16 to 66 years; male gender percentages of 44% to 75%). The performance of Dmax derived from ^18^F-FDG PET/CT was investigated in different lymphoma subtypes; the most representative lymphoma variant was DLBCL (*n* = 2296) [10,15,17,21,24,25,26,28,30], followed by HL (*n* = 455) [14,16,19,22,23]. There was a prevalence of advanced stage disease (stage III and IV) compared with early stage (stage I and II) (2101 cases compared with 520 (ratio 4:1)). In addition to Dmax, other semi-quantitative PET parameters were calculated, including SUVmax, MTV, TLG, and other texture features. Different software was used for the measurement of Dmax. LIFEX [10,15,16,17,19,26,27,28,29,31] and RaCat [22,23,24,25,30] were the most common. In some cases, Dmax was normalized by body surface area (BSA) and was called SDmax, changing the unit of measurement [15,17,18,21]. In half of the papers [10,15,16,19,20,26,27,28,29], a threshold of Dmax (or SDmax) was derived, with a very wide range (20–65.95 cm) (Table 3). These heterogeneities (lymphoma variants and index test key) did not allow us to perform a quantitative (meta-analysis) assessment. Three studies [14,17,23] investigated the reproducibility in the measurement of Dmax, comparing software and physician measurements [14] and different segmentation methods [17,23]. In all cases, the agreement and reproducibility were very high.

### 3.3. Risk of Bias and Applicability

**Figure 2:**
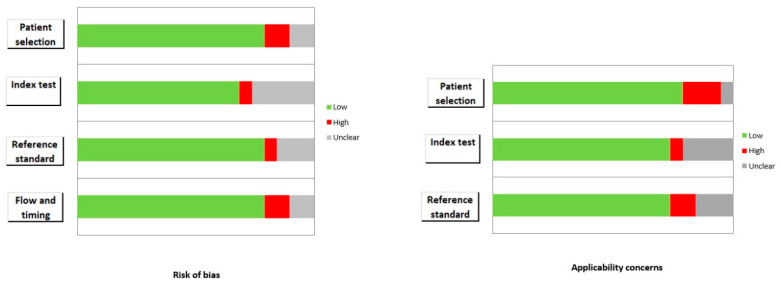
Summary of quality assessment according to the QUADAS-2 tool.

The overall assessment of the risk of bias and concerns about the applicability of the included papers according to QUADAS-2 are provided in Figure 2.

### 3.4. Prognostic Role

In most cases, Dmax demonstrated a significant correlation with progression-free survival (PFS) [10,15,16,19,20,21,24,25,26,27,28,29,30] and overall survival (OS) [10,15,16,21,26,27,28]; only in one study [18] was no significant association shown. Together with Dmax, the most frequent metabolic variable with a prognostic role was MTV [13,15,26,27], followed by interim metabolic response [19], TLG [20], and bone marrow ^18^F-FDG uptake [29]. In one article [22], Dmax was significantly associated with several blood sample markers, extracellular vesicles–microRNA (EV-mRNA), and thymus and activation-regulated chemokine (TARC) (Table 2).

## 4. Discussion

As ^18^F-FDG PET/CT has a cardinal role in the management of lymphomas, the measurements of semi-quantitative metabolic parameters, conventionally called radiomics, might become a non-invasive and useful way to derive independent biomarkers for personalized medicine. However, we currently suffer from significant methodological biases that may reduce the clinical translation of these parameters, especially related to their low reproducibility and validation. Many of these PET features (for example, SUV, MTV, and TLG) are strongly influenced by the operator, the type of scanner, and the acquisition and reconstruction parameters [32,33]. These limitations influence the possibility of routinely applying these variables.

In this scenario, Dmax may be a new biomarker, with fewer limitations in comparison with others. Dmax is a relatively simple 3D dissemination PET variable, which may intuitively represent the patient-based spatial migration feature of the disease. It is a very easy and fast to measure dimensional feature, and is less influenced by “technical” features. Dmax measures the distance between the centroids of two lesions; it is not impacted by the scanner or reconstruction/acquisition parameters, dissimilar to the other PET-derived features.

Another advantage of Dmax is the fact that it is not influenced by the operator as its measurement is automatic, and there are several existing software options that are able to perform this analysis with high accuracy and reproducibility. We can assume that Dmax may reflect tumor heterogeneity by directly visually representing the ability of tumor dissemination, with a diagnostic power superior to the traditional Ann Arbor staging.

This metric is different from the other imaging features that are more complex and difficult to translate into a clinical meaning. Unlike sophisticated radiomic features, often difficult to interpret from a biological point of view, Dmax intuitively reflects the spatial migration of the disease to different sites.

However, one of the limitations of Dmax is the unfeasibility to derive this variable in patients with a single lesion (stage 1). For example, Durmo et al. [19] excluded 14% of their initial population for this reason. Instead, in these patients, MTV, TLG, and other texture features may potentially be derived. An option could be to give a conventional Dmax value of 0 in patients with one lesion only, but this option needs to be explored. Another possibility could be to take the largest diameter of the single lesion.

Although it seems to represent a promising biomarker, Dmax needs further methodology refinement before any routine use.

For example, it is not clear if this parameter is affected by the height and/or body composition of the patient. In most papers, Dmax was not normalized to body size; however, in four articles [15,17,18,21], it was normalized by BSA, showing a better performance compared with a classical Dmax. The harmonization and sharing of the parameter definition should be the first step, allowing a more precise comparison and reproducibility of the results between different studies as well as the generation and validation of prognostic models with good accuracy that may also help treatment tailoring in routine clinical practice.

In many studies, Dmax was not the only feature with a prognostic role in the management of lymphomas, but was combined with other metabolic parameters. Amongst them, MTV was frequently significantly associated with survival [14,15,26,27]. For these reasons, Dmax and other volume-derived PET metrics may have a complementary role in outcome predictions. Moreover, MTV measurements may not have a substantial impact on Dmax because rare variations were obviously observed in the centroids of lesions with the different lesion sizes. However, Dmax and MTV must be correlated with clinical or biological data and validated in larger cohorts for the purpose of guiding clinical practice.

Another open issue is to set a threshold of Dmax to predict survival and better stratify patients. In the literature, we found many different cut-off values related to the population sample, including the lymphoma variant and methods to measure Dmax. This also needs clarification and a shared consensus. Moreover, preliminary evidence showed a strong correlation between Dmax and microenvironmental components of disease studied with gene expression profiling [19] and blood sample markers [22].

In addition to the baseline PET features, the interim and end-of-treatment PET findings were also studied, with a positive impact on prognosis [1,2,34]. Deauville scores and Lugano criteria were introduced in 2009 [35], and are based on the application of a five-point scale using the mediastinum and liver activity as the reference standard. These scores have been recommended for reporting in both interim and end-of-treatment PET for HL and several NHLs [1,2].

Some evidence in favor of the prognostic role of the combination of interim PET results and Dmax are available [19], but more robust data are needed.

In only one study based on mantle cell lymphoma—a particular lymphoma variant [18]—Dmax showed no prognostic role, but this work was the only paper. On the other hand, the only metabolic feature that showed a prognostic impact was MTV.

Lastly, we recognized that the main limitation of this systematic review was related to the clinical and methodological heterogeneity of the included studies. Therefore, we did not provide a quantitative synthesis through a pooled analysis. However, we followed a strict methodology to guarantee transparency and reproducibility; furthermore, the main findings reported in this evidence-based article could be very useful for suggesting further studies on Dmax in patients with lymphomas.

## 5. Conclusions

Overall, the available literature data on Dmax in lymphomas are limited and heterogeneous.

Dmax is a parameter that represents tumor dissemination, and has a strong prognostic role in different lymphoma variants. A model based on a combination of Dmax and other metabolic features such as MTV or Deauville scores may improve the prognostic value of PET and could guide individualized treatment.

The results of this systematic review need to be further evaluated in other large cohorts and compared with existing prognostic models to overcome the limitations of current clinical prognostic indicators in lymphomas.

## Figures and Tables

**Table 1:** Studies’ general information.

First Author	Year	Country	Study Design	Funding Sources
Cottereau, A.S. [10]	2020	France	Retrospective	None declared
Weisman, A.J. [14]	2020	USA	Retrospective	GE Healthcare; National Institutes of Health to the Children’s Oncology Group (U10CA098543), Statistics & Data Center Grant (U10CA098413), NCTN Operations Center Grant (U10CA180886), NCTN Statistics & Data Center Grant (U10CA180899), QARC (CA29511) IROC RI (U24CA180803); and St. Baldricks Foundation
Cottereau, A.S. [15]	2021	France	Retrospective	None declared
Zhou, Y. [16]	2021	China	Retrospective	None declared
Cottereau, A.S. [17]	2021	France	Retrospective	None declared
Vergote, V.K.J. [18]	2022	Belgium	Retrospective	None declared
Durmo, R. [19]	2022	Italy	Retrospective	GRADE Onlus; Associazione Italiana per la Ricerca sul Cancro; Italian Ministry of Health Ricerca Corrente Annual Program 2023
Li, H. [20]	2022	China	Retrospective	National Natural Science Foundation of China (No. 81771866).
Ceriani, L. [21]	2022	Switzerland	Prospective	Ente Ospedaliero Cantonale, Grant/Award Number: ABREOC 22008-262; Amgen; Oncosuisse, Grant/Award Number: OCS-02270-08-2008
Drees, E.E.E. [22]	2022	The Netherland	Retrospective	The Dutch Cancer Society, Grant/Award Number: KWF-5510; Cancer Center Amsterdam Foundation, Grant/Award Number: CCA-2013; Technology Foundation STW, Grant/Award Number: CANCER-ID
Driessen, J. [23]	2022	The Netherland/USA	Retrospective	None declared
Eertink, J.J. [24]	2022	The Netherland	Prospective	Dutch Cancer Society (# VU 2018–11648)
Eertink, J.J. [25]	2022	The Netherland	Prospective	Dutch Cancer Society (# VU 2018–11648)
Girum, K.B. [26]	2022	France	Retrospective	None declared
Gong, H. [27]	2022	China	Retrospective	None declared
Jo, J.H. [28]	2023	Korea	Retrospective	None declared
Xie, Y. [29]	2023	China	Retrospective	None declared
Eertink, J.J. [30]	2023	Netherland	Retrospective	None declared

**Table 2:** Patients’ main features and clinical results.

First Author	N Pts	Lymphoma Variant	Early (I–II)/Advanced (III–IV) Stage Acc Ann Arbor	M:F	Median Age (Range)	Main Results
Cottereau, A.S. [10]	95	DLBCL	0:95	53:42	46 (18–59)	Dmax was significantly associated with PFS and OS. The combination of MTV and Dmax helped to stratify patients
Weisman, A.J. [14]	100	HL	0:100	60:40	15.8 (5.2–21.4)	Moderate reproducibility in the Dmax measurement between fully automated software and physicians
Cottereau, A.S. [15]	290	DLBCL	26:264	170:120	Nr (60–80)	SDmax was significantly associated with PFS and OS. The combination of MTV and SDmax helped to stratify patients
Zhou, Y. [16]	65	HL	36:29	45:20	29 (8–72)	Dmax was significantly associated with PFS and OS
Cottereau, A.S. [17]	290	DLBCL	26:264	170:120	Nr (60–80)	Comparison of different ways to calculate dissemination features
Vergote, V.K.J. [18]	83	MCL	12:71	62:21	66 (58–72)	Dmax was not associated with prognosis
Durmo, R. [19]	155	HL	77:78	79:76	Nr	Dmax was significantly associated with PFS. Dmax and interim metabolic treatment response helped to stratify patients
Li, H. [20]	126	FL	22:104	63:63	53 (21–76)	Dmax and TLG were significantly associated with PFS
Ceriani, L. [21]	240	DLBCL	104:136	119:121	Nr	SDmax was included in a radiomics model with a prognostic value
Drees, E.E.E. [22]	30	HL	Nr	Nr	36 * (18–66)	Blood-based markers, EV-miRNA, and sTARC were moderately related to dissemination features
Driessen, J. [23]	105	HL	Nr	47:58	30 (13–66)	Good reproducibility of Dmax between 6 different segmentation methods
Eertink, J.J. [24]	317	DLBCL	51:266	161:156	65 (23–80)	Dmax_bulk_ was one of the best predictors of treatment outcome
Eertink, J.J. [25]	296	DLBCL	48:248	152:144	65 (55–72)	Dissemination features were the best predictors of progression
Girum, K.B. [26]	382	DLBCL	Nr	207:175	62.1 * (34–73)	Dmax was significantly associated with PFS and OS. The combination of MTV and Dmax helped to stratify patients
Gong, H. [27]	81	AITL	5:76	53:28	63	Dmax was significantly associated with PFS and OS. The combination of MTV and Dmax helped to stratify patients
Jo, J.H. [28]	63	DLBCL	26:39	28:35	57.3 * (21–87)	Dmax and end-of-treatment metabolic treatment response were significantly associated with TTP
Xie, Y. [29]	95	PTCL	10:85	59:46	64 (16–84)	Dmax and bone marrow biopsy were significantly associated with PFS and OS
Eertink, J.J. [30]	323	DLBCL	77:246	185:138	63 (53–71)	Baseline radiomics features were significantly associated with PFS

*: mean; M: male; F: female; HL: Hodgkin lymphoma; DLBCL: diffuse large B-cell lymphoma; FL: follicular lymphoma; AITL: angioimmunoblastic T-cell lymphoma; PTCL: peripheral T-cell lymphoma; MCL: mantle cell lymphoma; Nr: not reported; PFS: progression-free survival; OS: overall survival; TTP: time to progression; MTV: metabolic tumor volume; TLG: total lesion glycolysis; SDmax: Dmax normalized by body surface area.

**Table 3:** Main technical features.

First Author	PET Features	Software	Dmax Cut-Off	Dmax Median
Cottereau, A.S. [10]	SUVmax, MTV, TLG, Dmax_patient._ Dmax_bulk_, SPREAD_bulk_, and SPREAD_patient_	LIFEx	45 cm	45 cm
Weisman, A.J. [14]	SUVmax, MTV, TLG, SA/MTV, and Dmax	Deepmedic	Nr	Nr
Cottereau, A.S. [15]	MTV, Dmax, and SDmax	LIFEx	47 cm for Dmax 0.32 m^−1^ for SDmax	42 cm for Dmax 0.23 m^−1^ for SDmax
Zhou, Y. [16]	SUVmin, SUVmax, SUVmean, SUVpeak, SUVst, MTV, TLG, Dmax, histogram-derived features, shape-derived features, and texture features	LIFEx	57.4 cm	Nr
Cottereau, A.S. [17]	SDmax	LIFEx	Nr	Nr
Vergote, V.K.J. [18]	SUVmax, SUVmean, SUVpeak, MTV, TLG, Dmax, and SDmax	MIM	Nr	0.6 m for Dmax 0.3 m^−1^ for SDmax
Durmo, R. [19]	MTV, TLG, and Dmax	FIJI and LIFEx	20 cm	20 cm
Li, H. [20]	SUVmax, MTV, TLG, and Dmax	R	56.73 cm	64 cm
Ceriani, L. [21]	SUVmax, SUVmean, MTV, TLG, SDmax, and texture features	PyRadiomics Python	Nr	Nr
Drees, E.E.E. [22]	SUVmax, SUVpeak, MTV, TLG, Dmax_Patient_, Dmax_Bulk_, Spread_Patient_, and Spread_Bulk_	RaCat	Nr	Nr
Driessen, J. [23]	SUVmax, SUVmean, SUVpeak, MTV, TLG, and Dmax	RaCat	Nr	Nr
Eertink, J.J. [24]	SUVmax, SUVmean, SUVpeak, MTV, TLG, Dmax_patient_, Dmax_bulk_, SPREAD_bulk_, SPREAD_patient_, and texture features	RaCat	Nr	Nr
Eertink, J.J. [25]	SUVmax, SUVmean, SUVpeak, MTV, TLG, Dmax_patient_, Dmax_bulk_, SPREAD_bulk_, SPREAD_patient_, and texture features	RaCat	Nr	Nr
Girum, K.B. [26]	MTV and Dmax	LIFEx	59 cm	98 cm for REMARC 116.4 cm for LNH073B
Gong, H. [27]	MTV and Dmax	LIFEx	65.7 cm	66.4 cm
Jo, J.H. [28]	SUVmax, SUVmean, MTV, TLG, and Dmax	LIFEx	27.5 cm	Nr
Xie, Y. [29]	SUV, MTV, TLG, and Dmax	LIFEx	65.95 cm	69.3 cm
Eertink, J.J. [30]	SUVmax, SUVmean, SUVpeak, MTV, TLG, and 12 dissemination features	RaCat	Nr	Nr

SUV: standardized uptake value; MTV: metabolic tumor volume; TLG: total lesion glycolysis; SA: surface area; SDmax: Dmax normalized by body surface area.

## Data Availability

Not applicable.

## Supplementary Materials

The following supporting information can be downloaded at: https://www.mdpi.com/article/10.3390/cancers15092494/s1, Table S1: PRISMA checklist [36].
